# Decentralized and Dynamic: The Sociomaterial Flow of Peer-Led Learning in Digital Spaces

**DOI:** 10.5334/pme.2107

**Published:** 2025-11-06

**Authors:** Siew Ping Han, Nevin Chua Yi Meng, Emmanuel Tan Chee Peng, Jennifer Cleland

**Affiliations:** 1 Physiology/Medical Education, Lee Kong Chian School of Medicine, Nanyang Technological University, Singapore; 2 Lee Kong Chian School of Medicine, Nanyang Technological University, Singapore; 3 Medical Education, Lee Kong Chian School of Medicine, Nanyang Technological University, Singapore

## Abstract

**Introduction:**

Social media has transformed medical education by facilitating learner-led digital communities that co-construct knowledge. Previous studies position it as a passive information delivery tool. This fails to capture complex relationships between learners and technologies that may create new potentials for learning, teaching, and participation in the educational process. Our aim was to explore the interactions and patterns of information flow emerging within “What is, this is” (WITI), a social media-based peer teaching community of medical students.

**Methods:**

This was a qualitative study using a digital ethnography approach. We collected multiple sources of data comprising screenshots of WITI pages (n = 35), observations of participant interactions on WITI, and individual semi-structured interviews (n = 14). Data were analysed using abductive thematic analysis with relational transfer as a theoretical framework.

**Results:**

We identified three themes: 1) engagement in a connective space; with 2) decentralization of knowledge and authority; leading to 3) co-production of knowledge as a community. We found that learners actively shaped the use of social media technology and learner practices were patterned by social media affordances in a dynamic and symmetric relationship. This gave rise to a decentralized, collaborative and self-regulated knowledge-sharing digital community wherein the roles of teachers and learners were fluid and non-exclusive.

**Discussion:**

Our study provides insight into new and potentially disruptive learning practices and teacher-learner relationships within student-led digital learning spaces. Our findings highlight the potential of social media to support peer teaching amongst medical students, and the need to reimagine the role of the medical educator as a guide for, and collaborator with, students.

## Introduction

The growth in the use of technology in education has been accompanied by interest in researching the impact of digital technologies such as laptops and tablets, online learning platforms and the Internet, computer software, videoconferencing on learning, teaching, and education.

One technology which is of particular interest in this space is social media [1,2]. Social media encompasses online platforms that facilitate creation, curation and sharing of content between participants in a user-created social network characterized by the multi-directional flow of information [3]. It has become an increasingly popular online learning resource for medical students, with approximately half of them using it daily to augment their learning [4,5] and with users deciding when and how they learn [6].

Previous studies of social media in medical education tend to focus on its use by physicians as a teaching tool to disseminate knowledge [6,7,8]. This approach positions social media as a passive instrument wielded by physicians to deliver information to learners, like the traditional classroom where the teacher controls the content and direction of information flow [9]. Similarly, studies to date have positioned social media as a neutral tool rather than an active constituent of the learning process [10,11].

This positioning fails to acknowledge that, first, many social media learning spaces are learner-led [e.g. 12]; second, that learners in digital learning communities share and construct knowledge together [6,13]; and third, that the interactions between human actors and digital technologies introduce new mobilities negotiated through spatial re-orderings of information flow [14]. Users and social media are constitutively entangled in an integrative entity wherein each enacts the other in practice [15]. In other words, the relationship between social media, other digital technologies and learners is symmetric, one of mutual constitution and coevolution [16,17,18]. Exploring these new student-led social media-based digital learning spaces entails a shift to a relational approach, to understanding learning spaces as dynamic and fluid products of relations between social, textual (discourses) and material factors [19]. In this framework, “learning and knowledge do not reside in individuals; they circulate in relationships, which in turn can coalesce; that is, form seemingly fixed points and foundations” [20].

Viewed through a practice lens, social media affordances arise from material and social practices through repeated interactions between users and platforms, resulting in emergent technology structures that shape organizational relationships and patterns of interactions [21,22]. The spaces and objects inherent in educational social media, and how these dynamically interact with people, may have consequences in terms of changing the relationships between learners and teachers, and creating new potentials for learning, teaching, and participation in the educational process. They are thus worthy of our attention.

Given this, our aim was to identify and examine how interactions and patterns of information flow emerged from student use of a social media-based question bank [12]. Our specific research question was: How do students interact with the features of social media in a digital community for peer teaching and learning? Working from the sociomaterial perspective, we wanted to know ‘how materials (in this case, social media learning spaces) participate in practice and what is thereby performed’ [23, p. 28]. We adopted Mulcahy’s relational view of learning spaces and learning transfer [19,20] as a sensitizing concept to guide our inquiry. Our aim was to demonstrate the ‘mangle of practice’ [24] between social media and people: in other words, what practices and experiences are generated through the interplay between learners and a social media learning resource.

## Methods

This was a qualitative study. We drew upon the principles of digital ethnography, a research approach that correlates multiple data sources to build detailed, comprehensive descriptions of daily digital practices [25]. Digital ethnography can be defined as research ‘on online practices and communications, and on offline practices shaped by digitalization’ [26, p.57]. Digital ethnography is ontologically aligned with sociomateriality.

Unlike traditional ethnography, brief immersion in the field is acceptable in digital ethnography because the nature of digital data and research tools allows the researcher to trace long-term engagement in a particular topic area using digital footprints.

### Study context

Our focus was a student-created, student-managed social media-based question-setting and answering community known as ‘What is, this is’ (WITI) [12] based within a medical school in Singapore. WITI was designed to promote retention and recall of information through repeat testing, give immediate feedback to identify knowledge gaps and provide explanations for correct and incorrect options. WITI spanned all five years of the medical degree (MBBS) programme, with students in each year setting questions for their own cohort. Question topics usually reflected the content covered in the formal curriculum. WITI student administrators posted questions in single best answer format, typically on a weekly basis during termtime. Students attempted these questions using the ‘poll’ function and received immediate feedback on the correctness of their choices. Detailed explanations for the answers were provided in a subsequent post soon after or in the ‘comments’ section of the user interface.

The initial iteration of WITI was implemented on the Instagram platform, leveraging its ‘poll’ feature and the capability to post ‘stories’. At its peak, the WITI Instagram page garnered a user base of 578 individuals (out of a medical student population of approximately 700 at that time). In 2022, WITI transitioned to the Telegram messaging platform, where channels were segmented by academic year and specific topic areas to enhance the relevance and utility of question dissemination for each student cohort. Typically, each Telegram channel attracted between 120 and 130 subscribers out of the approximately 160 students in that respective year, with the highest recorded subscription rate being 165 of 168 students, and the lowest 112 of 163.

In this paper, we refer to the students who posted questions and the students who answered them as setters and users respectively, although the two roles were not mutually exclusive.

### Data collection

We used multiple sources of evidence and layered data collection strategies to familiarize ourselves with both human and non-human data sources, and the interplay between the various actors.

We first obtained an overview of the digital and organizational structure of WITI from its creators and question setters (n = 5) via email correspondence. We then used observation and interviews as our main forms of data collection.

Initially, for approximately one year (mid-2022 to mid-2023), team member NCYM passively observed the interactions within WITI in the capacity of a WITI ‘user’ without keeping formal field notes. Following this, from June 2023 to February 2024, NCYM observed WITI from the perspective of an ‘observer’, looking for specific patterns and interactions. He checked the WITI channel twice a week (Tuesdays and Fridays, 12pm) and noted his observations. Over this time, NCYM observed at least three versions of WITI, with the first version hosted on Instagram and later versions transiting to Telegram. He collected 35 screenshots of WITI over a four-month period (June 2023 to September 2023). These snapshots in time served as visual records of the human-human and human-platform assemblages within the digital learning space [27,28].

Drawing on our email discussions with WITI creators and setters, plus our previous study of WITI and relevant literature [3,12,29], we designed a semi-structured interview schedule to explore different stakeholders’ roles within WITI and how they interacted with both the social media platform and with each other. The interview schedule (supplementary information) ensured consistency across interviewers and interviews while providing flexibility to explore experiences unique to each interviewee.

Team members (SPH and ETCP) first interviewed the two WITI creators and three WITI setters. Using a snowball sampling approach [30], we asked them to direct us to students who frequently answered questions on WITI (WITI users, n = 3). This gave a total of eight interviews carried out between April-November 2022.

It was clear from these interviews that WITI was a dynamic entity, and so we followed up the original interviews with an additional six interviews between February-March 2025. As before, the interviewees included WITI setters (n = 2) and users (n = 2) but we also included two WITI non-users (n = 2).

In total, we carried out fourteen interviews (419 minutes of interview data, median interview length of 25 minutes) with ten male and three female medical students from Years 2–4 of a five-year degree program, plus one male alumnus (one of the original WITI creators).

### Data analysis

Interviews were digitally audio-recorded with full consent for later transcription and then anonymised throughout the transcription process.

Data were analyzed by abductive analysis [31], where we used the theory of relational transfer as a broad lens to guide analysis while at the same time being open to new knowledge from the empirical findings [32].

After familiarisation with the interview data, screenshots and observations, team members SPH and NCYM organised the data, repeatedly revisiting it to identify patterns and connections in relation to the research question. They then performed thematic analysis [33] using Mulcahy’s theory of relational transfer as an analytical lens to identify social, material and textual elements and the relationships between them within the sociomaterial assemblage of a learning space [20]. Through this process, and in keeping with the principles of abductive analysis [31], we adjusted our framework and adopted a more specific practice lens [22] to focus specifically on how social media affordances were actualised through functional-oriented practices as described by Namisango et al., 2023. This included socialising (building associations between an organisation, its participants and its content), information sharing (transmission of information resources through one- and two-way communication) and visibility (an organisation’s presence, activity and communication) [21]. Any coding disagreements were discussed and resolved within the research team.

### Reflexivity

In line with sociomaterial approaches, we positioned ourselves as active agents in the assemblage [34,35]. We considered and reflected upon our different relationships with WITI. As a medical undergraduate and WITI user, NCYM was an ‘insider’, a medical student with direct access to the WITI space, enmeshed with WITI as a user as well as being familiar with how his peers interacted within and with WITI. The other authors were faculty members and therefore ‘outsiders’ to WITI. We all interacted with different forms of social media for personal and educational purposes. More generally, we had different positions on health professions education research and sociomateriality theory related to our training (e.g., medicine, psychology, biomedical science, law), theoretical interests and knowledge, and research experience (e.g., JC has used sociomaterial theory in research previously [36]).

### Ethical approval

Ethical approval was obtained from the Nanyang Technological University Institutional Review Board (IRB-2020-07-018).

## Results

We identified three main inter-related themes:

1. Engagement in a connective space; with
2. Decentralization of knowledge and authority; leading to
3. Co-production of knowledge as a community

### Engagement in a connective space

We found that social media platforms, in combination with online resources, transformed the sociomaterial arrangements of learning spaces, learners and educators. WITI was entirely created and run by students. Question setters drew upon online resources such as Google and online medical question banks to set questions beyond the scope of their existing knowledge. Such student-led practices were enabled by the connectivity of social media platforms which facilitated free, convenient and instantaneous information transfer and sharing between users.

WITI designers were mindful of student needs and preferences, with WITI setters stating that their ‘priority has always been accessibility’. Thus, although WITI was initiated on Instagram, as the student population became more active on Telegram, the WITI setters decided to shift from Instagram to Telegram. In other words, user activity was a key factor in shaping the choice of platform and subsequent technological structures.

‘[W]e just decided that overall Telegram is more accessible than Instagram because not everyone checks Instagram all the time also, whereas on Telegram, people are usually already talking to other people and checking their school groups and all that.’ (Participant 5)

**Figure 1:**
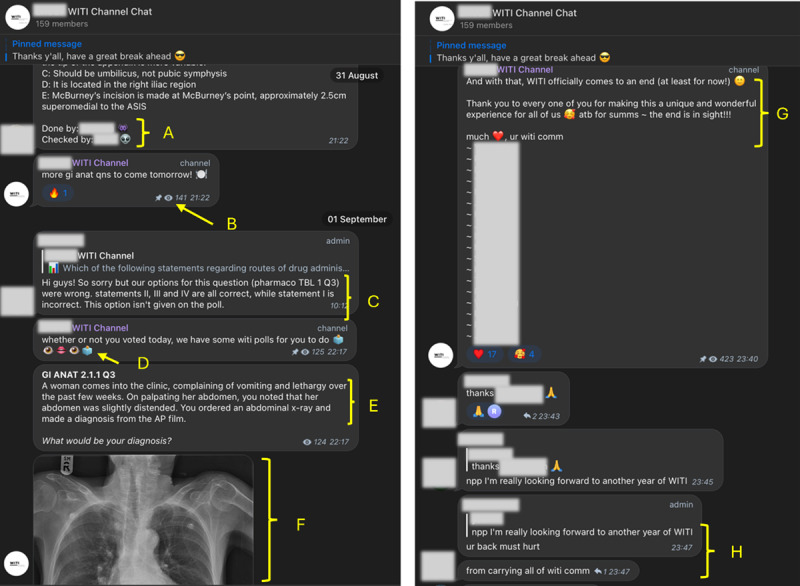
Screenshots of WITI channel (with identifiers blurred out) demonstrating how students utilize digital affordances provided by social media-based learning spaces. A) Vetting system where the students who wrote and checked each question were named. B) Number of students who engaged with each post could be monitored. C) Correction to previous question was made within a day. D) Frequent use of emojis. E) Formal phrasing of medical question. F) Convenient insertion of images. G) Encouraging messages to create a supportive environment. H) Casual language with text abbreviations commonly used by students.

Telegram features enabled WITI to exhibit a high level of social interactivity. In between formally worded questions, students posted hints, updates and words of encouragement to each other, with frequent use of emojis and text abbreviations (Figure 1). WITI setters were able to monitor and respond to reactions and replies in real time, allowing them to make frequent and timely adjustments in response to feedback from users. The frequent, positive and dialogical interactions made possible by Telegram boosted engagement and created a sense of community in which student participation and ownership was valued. Indeed, both setters and users stated that the fact that WITI was created by students to serve peers was its ‘greatest strength’.

Some students preferred to use WITI over commercial digital medical resources available to all students via paid subscription by the medical school. Conversely, other students did not engage with WITI or disengaged because they felt the explanations provided for answers were insufficient or they preferred using medical school-provided resources.

The fact that WITI took place on platforms that students used for social interactions gave it a very strong socialization element. This mixing of “business and pleasure” was liked by some but disliked by others: ‘On Telegram it’s easy to get distracted when friends are texting, so it doesn’t feel like a study environment’ (Participant 14).

### Decentralisation of knowledge and authority

WITI setters saw themselves as learners not experts, expressing doubt as to whether they ‘were even knowledgeable enough to set questions’. They saw question setting and its associated research as a way to enhance their own learning.

**Figure 2:**
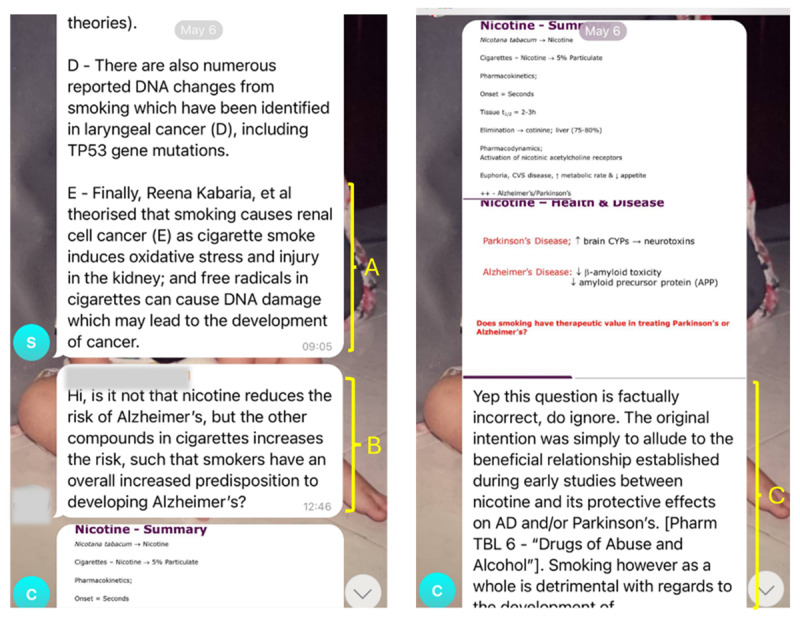
Screenshot of message thread between setter posting an answer with detailed explanations, including citations from the literature (A) and user providing an alternative answer (B). Setter then acknowledged the factual inaccuracy and provided further explanation (C). Note that this exchange occurred within a day.

In the absence of a central and authoritative source of knowledge, trust in the accuracy of content was instead established through a mutual vetting system wherein setters checked and approved each other’s questions before posting them on the WITI channel (Figure 1A). Setters cited publications to substantiate the accuracy of their answers and explanations, thereby leveraging upon the authority of academic publications in the absence of their own (Figure 2A).

‘[O]ur workflow is that a few people will set the questions, we’ll get a different person to fact check the questions because accuracy is important to us, we don’t want to post a question and then realise there’s a bit of factual errors in the question.’ (Participant 3)

Likewise, users took the questions and explanations ‘with a pinch of salt’ and trusted that errors would be identified and rectified within the WITI community.

‘[W]e have student-made questions, there may be errors in the questions, so that is one thing, although it’s quite rare. But I think there have been errors in the WITI questions for which someone will point out to them, then they’ll just do a correction, which is alright.’ (Participant 6)

Setters welcomed user feedback on their questions, candidly acknowledging gaps in their own knowledge and recognizing users as equal contributors to improving content (Figure 2B, 2C). Inaccuracies were quickly picked up and rectified to counter misinformation. In this way, authority was decentralized across participants and the online resources used to support their explanations.

This decentralized control of information flow was supported by the Telegram interface design. All users on the channel had equal access to post and receive information. All posts had the same text and visual format, without distinction between setters and users. The limited space on a typical mobile phone screen coupled with the frequency of new posts meant that no individual dominated the interface. IDs were small and positioned at the edge of the interface so that the focus remained on the content and not on who was posting it. Thus, human-human and human-technology interactions were enmeshed to distribute the work of knowledge production across the entire WITI assemblage.

### Co-production of knowledge as a community

Willingness to set questions for their peers was the sole criterion for becoming a WITI setter. WITI setters built their knowledge and skills through self-directed learning as well as input from seniors, fellow setters and WITI users. They attempted questions from other setters and were therefore also WITI users. Thus, WITI setters assumed the dual roles of learner and educator, often occupying both roles simultaneously as they learnt through question setting and answering.

‘I think it’s definitely made me more sure of the content that I cover in the questions and when somebody asks me about a question I’ve set and they’re not sure why the answer is a certain option and not something else, if I’m able to explain it to them, I think that means that I have understood the content.’ (Participant 5)

This blurring of distinction between learner and educator arose from the undirected connectivity of social media, which fostered dialogue and reciprocal exchange of ideas. Meanings were negotiated between multiple participants, who were free to agree or disagree with as well as post content. There was a sense that the students were learning from and teaching each other. Even non-users were highly supportive of WITI and appreciated the efforts of WITI setters, even if they did not actively participate on the platform.

‘So I think, having like a whole batch, you know, having eyes on these particular questions and helping to improve them, I think, is quite good for learning for everyone involved.’ (Participant 7)

WITI was dynamic: the WITI community was constantly acting on the social media platform to tailor it to their learning goals. For example, in the clinical years, each cohort is split into three streams covering different medical specialties at different times. The WITI channel was similarly split into three channels so that questions could be aligned with the content covered in each stream in a timely manner.

‘So there’s actually three different like WITI, like platforms in a sense, for the different streams, ‘cause we would have covered different content and the content will just be like everything under that particular like posting that we’ve just done’ (Participant 8).

## Discussion

Our study shows that the traditional representational rendering of learning transfer, that knowledge is separate from the material world and is transferred from teacher to student [20], is less relevant in collaborative digital learning environments where mobile technologies and devices shape educational practices [37] and learning is distributed across the entire assemblage of actors [34].

The adoption of social media technology freed learning practices from the constraints of traditional classroom arrangements, where existing patterns of information flow, controlled and limited by the teacher, are stabilized and maintained by institutionally based routines and resources [38]. Instead, WITI, situated on a social media platform and the internet, accessible via mobile phones 24/7, provided students with the space and tools to gather and distribute knowledge without institutional involvement.

In the non-hierarchical, casual, dynamic and student-led learning space of WITI, students acted as knowledge gatekeepers rather than content experts, setting up systems to facilitate and regulate the sharing of information between their peers. Information was instantly and freely shared across the entire community, with setters providing questions and answers and users providing feedback on their quality and accuracy. This meant that all students were able to contribute to knowledge transfer within the WITI assemblage. Moreover, teaching and learning within WITI were deeply entangled with the materials and technologies of social media, with students using the platforms’ digital affordances to exchange knowledge, socialize, and provide, collect and respond to feedback. In other words, knowledge was produced through relational transfer via the interactions between humans (student), as well as human and non-human (internet) entities [39].

While our work aligns with other studies of informal peer or near-peer teaching amongst medical students, which have described how peer teachers learn through teaching, assume roles traditionally assumed by faculty teachers and are perceived as being more interactive and accessible than faculty teachers [reviewed in 40], it provides new insight by highlighting how intermingling of WITI participants and the social media platform generates new patterns of practice [21]. WITI had a very strong socialization element, since it took place on a platform that students engaged with regularly for social interactions. Information was freely shared across the entire community, with setters providing questions and answers and users providing feedback on their quality and accuracy. Interactions were highly visible, thus building trust and engagement amongst participants.

One of the challenges of near-peer teaching and digital learning communities is that responsibility for judging the accuracy of information falls upon individual users and there are limited means of regulating educator credentials [3,40]. WITI circumvented this by establishing a question vetting system, an approach akin to crowdsourcing, which harnesses the collective intelligence of a community and decentres the individual “to elevate the logic of the many” [41].

Connectivity on social media is not an inherent attribute but rather an ongoing process achieved by intermingling human and digital agencies [42]. In our context, Telegram was originally used by students for casual group communications. The students then reconfigured the relationship between themselves and the platform by creating a social media-based community for aiding examination preparation. In turn, social media shaped user behaviours and practices by encouraging sociality and engagement through features such as emojis, flexible access and ease of posting information [43].

Our case study demonstrates how the entanglement of social media affordances and medical student-led knowledge-sharing communities created new digital learning spaces and disrupted traditional pedagogical roles, relationships and practices [49]. WITI exemplifies the shadow curriculum, a curriculum that functions completely or mostly independently of formal curricula, organizational structures and relationships between faculty and students [44].

However, such student-run digital learning spaces come with new sets of challenges. The bite-sized learning content offered by the internet and social media may appeal to learners, but often information integrity and the complexity of clinical teaching are sacrificed for brevity and simplicity [45]. Medical students may lack the critical thinking skills or clinical experience to identify unreliable, inaccurate or incomplete information, even as a self-regulating community [46]. In the absence of a central, authoritative source of knowledge, students might reinforce each other’s misconceptions, especially in an online world where health misinformation is all too prevalent, especially on social media [47]. Further, medical students tend to be examination-oriented and focus on learning ‘testable’ content rather than the skills and knowledge most relevant to actual clinical practice [48]. Without guidance and role modelling from experienced clinicians, wholly student-run learning spaces risk becoming overly fixated on superficial learning approaches and factual recall.

As the ‘digital undertow’, below-surface waves that are invisible yet powerful [49], disrupts institutional- and teacher-centric education practices, new roles have emerged for medical educators as knowledge gatekeepers, acting as the ‘guide on the side’ to support student learning activities. Their responsibilities should shift towards helping students develop higher-order clinical reasoning and critical analysis skills to better understand and assimilate readily available online medical information. Educators may need to integrate their teaching practices with new digital learning spaces in which the teacher-student hierarchies are reduced or non-existent. Shifting to such collaborative rather than control roles may not be comfortable for educators.

Student-run digital learning spaces are likely to become an increasingly important medical education resource due to the growing availability and affordability of digital tools for information sharing. More research is required to investigate and understand how human and non-human interactions are reshaped in these complex and dynamic assemblages.

### Strengths and limitations

One obvious strength of this study is its strong theoretical grounding, using Mulcahy’s conception of relational transfer and Orlikowski’s practice lens [20,22]. We fill a significant gap in the literature about learning spaces by focusing on student-run, digital learning spaces which are usually ‘hidden’ from medical education researchers because they operate independently from institutional involvement.

In terms of trustworthiness of the data, we ensured credibility by triangulating data (interviews, observations, screenshots), acknowledging our personal biases in the reflexivity section and including a team member (NCYM) who was a long-term WITI participant with prolonged engagement with the platform. Dependability was obtained through detailed documenting of the research process, confirmability by including team members with varied perspectives; and transferability by providing detailed descriptions of the study context and sample.

The presence of an ‘emic’ view in the research team is a strength of the study, providing access and insight into WITI’s authentic, inner workings. However, we acknowledge that this came from a single student, whose personal views may influence data collection and interpretation. We mitigated this by having frequent team discussions during data collection and analysis, as well as triangulating interview, image and textual data. Our number of interviews was relatively small but the research question was focused, our participants were ‘information-rich’ and we found that participants shared similar experiences and views [50]. We interviewed more males than females. To some extent this reflects the gender balance at our medical school. However, future research may usefully examine if there are gender preferences and patterns in relation to digital resource creation and use among medical students.

## Conclusion

Social media platforms enable medical students to set up new digital learning spaces which are collaborative, dynamic and non-hierarchical, enabling multidirectional and decentralized exchanges of information. These new patterns disrupt the traditional roles of teachers and institutions and, as such, lead to the need to re-conceptualize teaching practices within medical education.

## Additional File

The additional file for this article can be found as follows:

10.5334/pme.2107.s1 Supplementary Information. Interview schedule.
