# Supplementary material for: Decentralized and Dynamic: The Sociomaterial Flow of Peer-Led Learning in Digital Spaces
Source: Perspect Med Educ. 2025 Nov 6;14(1):725–35. doi: 10.5334/pme.2107 (PMC12594076; doi:10.5334/pme.2107)
Supplement: Supplementary Information. — Interview schedule. [file pme-14-1-2107-s1.pdf]

Supplementary Information:

Semi-structured interview questions for WITI creators/setters

How did WITI come about? What was the original aim?

How has WITI evolved over the years in terms of its aims, design (how it works) and organisation (how the people involved work together)?

To what extent do you feel WITI has achieved its aims?

What challenges have you met along the way (with respect to WITI) and how were they overcome?

How useful do you feel WITI is to the general student population?

Semi-structured interview questions for WITI users

How have you made use of WITI? (frequency, when during the term)

Do you feel WITI has helped in your learning, and if so, how?

How does WITI compare to other question banks/ learning resources that are available to LKC students?

How has WITI evolved over the years in terms of its design? How effective do you find the various platforms used?

How useful do you feel WITI is to the general student population?

Have you provided feedback on WITI? If so, in what way?
